# Clinical characteristics and risk factors for septic shock in patients receiving emergency drainage for acute pyelonephritis with upper urinary tract calculi

**DOI:** 10.1186/1471-2490-12-4

**Published:** 2012-03-13

**Authors:** Yoshiyuki Yamamoto, Kazutoshi Fujita, Shigeaki Nakazawa, Takuji Hayashi, Go Tanigawa, Ryoichi Imamura, Masahiro Hosomi, Daiki Wada, Satoshi Fujimi, Seiji Yamaguchi

**Affiliations:** 1 Department of Urology, Osaka General Medical Center, Osaka, Japan; 2 Department of Emergency, Osaka General Medical Center, Osaka, Japan

## Abstract

**Background:**

Acute pyelonephritis (APN) is a common complication of ureteral obstruction caused by urolithiasis, and it can be lethal if it progresses to septic shock. We investigated the clinical characteristics of patients undergoing emergency drainage and assessed risk factors for septic shock.

**Methods:**

A retrospective study was performed of 98 patients (101 events) requiring emergency drainage at our urology department for obstructive APN associated with upper urinary tract calculi from January 2003 to January 2011. Clinical characteristics were summarized, and risk factors for septic shock were assessed by logistic regression analysis.

**Results:**

Objective evidence of sepsis was found in 64 (63.4%) events, and 21 events (20.8%) were categorized as septic shock. Ninety-six patients recovered, but 2 patients died of septic shock. Multivariate analysis revealed that age and the presence of paralysis were independent risk factors for septic shock.

**Conclusions:**

APN associated with upper urinary tract calculi is a severe disease that should be treated with caution, particularly when risk factors are present.

## Background

Acute pyelonephritis (APN) is a common complication of ureteral obstruction caused by urolithiasis, which can be lethal if it progresses to sepsis or septic shock. APN is broadly divided into uncomplicated and complicated cases. Complicated cases are defined to have anatomical or functional abnormality of urinary tract, such as ureteral obstruction caused by urolithiasis. It has been estimated that 41% of patients with complicated APN develop severe sepsis or septic shock [1]. The clinical presentation of APN can vary widely from loin pain and a positive urine culture to fulminant sepsis with a severely ill patient. APN associated with upper urinary tract calculi sometimes requires emergency drainage via a ureteral stent or percutaneous nephrostomy. Previous studies on the clinical characteristics of sepsis associated with upper urinary tract calculi have shown that bacteremia is an indicator of severe APN [1], while old age and poor performance status are risk factors for emergency drainage [2]. Despite intensive management and emergency drainage, the mortality rate remains around 2% [2]. We retrospectively studied the clinical characteristics of patients with septic shock due to APN with upper urinary tract calculi and investigated risk factors for septic shock.

## Methods

A retrospective study was performed on 98 patients (a total of 101 presentations for APN) who required emergency drainage at our urology department for treatment of APN with upper urinary tract calculi from January 2003 to January 2011. The diagnosis of urolithiasis was based on clinical manifestations, urinary sediment, plain X-ray findings, ultrasound and computed tomography (CT) data. We examined the presence of hydronephrosis and/or renal insufficiency. In our department, we first try to perform ureteral stent insertion for the drainage of upper urinary tract by calculi under local anesthesia. In the case of the failure of stent insersion, we next perform percutaneous nephrostomy under local anesthesia. If APN with upper urinary tract calculi was diagnosed and emergency drainage (either retrograde instrumentation or percutaneous nephrostomy) was performed, patients were enrolled in this study. The diagnosis of acute pyelonephritis was based on clinical manifestations, body temperature, laboratory test results, and imaging data (plain X-ray, ultrasonography, and CT). Emergency drainage was selected if there was evidence of inflammation, such as high fever (axillary temperature > 38.0°C), a high white blood cell count, and an elevated C-reactive protein (CRP) level. The body temperature, mean blood pressure, and heart rate were always measured. The method of drainage, location and size of calculi, interval from the onset of symptoms to drainage, and duration of hospital stay were analyzed. The white blood cell count, platelet count, serum creatinine, CRP, and serum albumin at presentation were also assessed.

Patients were categorized into groups with or without septic shock and the clinical characteristics of the two groups were compared. Septic shock was defined as severe sepsis plus one of the following: mean blood pressure (BP) < 60 mmHg (< 80 mmHg for patients with known hypertension) after 40 ml/kg of saline or the need for dopamine (5 mg/kg/min) to maintain a mean BP > 60 mmHg (80 mmHg for prior hypertension) [3]. Severe sepsis was defined as sepsis associated with at least one of the following signs of organ hypoperfusion or organ dysfunction: abrupt change of mental status; abnormal electroencephalographic findings; platelet count < 100,000/ml or evidence of disseminated intravascular coagulation; or cardiac dysfunction on echocardiography [3]. Results are shown as median values. Univariate analysis was performed with Fisher's combined probability test or the Mann-Whitney U test. Independent predictors of septic shock were identified by multivariate logistic regression analysis. Variables entered into the model were age, sex, history of urolithiasis, presence of bacteremia, diabetes mellitus, hypertension, and psychosis, history of cerebral infarction (or hemorrhage or aneurysm), cardiovascular disease, presence of paralysis, and performance status (according to the Eastern Cooperative Oncology Group: ECOG). Statistical significance was defined as *p *< 0.05.

## Results

Ninety-eight patients required emergency drainage by percutaneous nephrostomy or ureteral stenting for the treatment of APN with upper urinary tract calculi on a total of 101 occasions. All 98 patients were hospitalized. Emergency drainage was performed a total of 1, 2, and 3 times in 96 patients, 1 patient, and 1 patient, respectively. All cases had hydronephrosis. Table 1 lists the characteristics of the patients and their calculi, as well as intervention and hospitalization data. The median age was 67 years (range: 29-90 years). Sixty-nine (68.3%) events occurred in females and 32 (31.7%) occurred in males. For 22 events (21.8%), calculi were located in the kidney, while calculi were in the ureter for 74 events (73.3%) and the location was unclear for 5 (5.0%). The median size of the stones was 9 mm (range: 1-78 mm). A case of stone in 1 mm diameter had a ureteral stenosis. Of the 101 events, 69 (68.3%) were associated with a positive urine culture and 36 (35.6%) with bacteremia.

**Table 1:** The characteristics of patient, calculi, intervention and hospitalization

		Total	No septic shock	Septic shock	*P *value
Characteristics	Events	101	80	21	

Age	Median (range)	67 (29-90)	64 (29-78)	74 (34-90)	< 0.001

Sex	Male	32 (31.7%)	27 (33.8%)	5 (23.8%)	0.441

	Female	69 (68.3%)	53 (66.3%)	16 (76.2%)	

Body mass index	Median (range)	22.4 (13.9-35.7)	22.8 (13.9-35.7)		

				22.3 (17.6-27.4)	0.414

Stone location	Right	48 (47.5%)	38 (47.5%)	10 (47.6%)	

	Left	47 (46.5%)	36 (45.0%)	11 (52.4%)	

	Bilateral	6 (5.9%)	6 (7.5%)	0 (0%)	

	Kidney	22 (21.8%)	15 (18.8%)	7 (33.3%)	0.229

	Ureter	74 (73.3%)	61 (76.3%)	13 (61.9%)	

	Unclear	5 (5.0%)	4 (5.0%)	1 (4.8%)	

Stone size (mm)	Median (range)	9 (1-78)	9 (3-78)	9(1-20)	0.327

	Unclear	14 (13.9%)	12 (15.0%)	3 (14.3%)	

Drainage	Ureteral stent insertion	90 (89.1%)	73 (91.3%)	17 (81.0%)	0.168

	Percutaneous nephrostomy	11 (10.9%)	7 (8.8%)	4 (19.0%)	

Culture	Positive urine culture	69 (68.3%)	53 (66.3%)	16 (76.2%)	0.441

	Bacteremia	36 (35.6%)	21 (26.3%)	15 (71.4%)	< 0.001

Intratracheal intubation		13 (12.9%)	4 (5.0%)	9 (42.9%)	< 0.001

Days from initial symptom to drainage	Median (range)	3 (0-38)	3 (0-38)	2 (0-16)	0.008

Hospital stay (day)	Median (range)	11 (1-171)	10 (1-171)	14 (1-95)	0.011

Mortality		2 (2.0%)	0 (0%)	2 (9.5%)	

Of the 101 events, 90 (89.1%) were managed by ureteral stenting and 11 (10.9%) were managed by percutaneous nephrostomy. Thirteen patients (12.9%) required endotracheal intubation because their respiratory status deteriorated. The median time from onset of symptoms to drainage was 3 days (range: 0-38 days) and the median hospital stay was 11 days (range: 1-171 days). Two patients died despite receiving emergency drainage.

Table 2 lists the laboratory data. The median white blood cell count was 12.9 × 1,000/μL (range: 0.7-38.3), the platelet count was 14.6 × 10,000/μL (range: 0.3-77.0), serum creatinine was 1.6 mg/dl (range: 0.5-11.6), CRP was 16.1 mg/dl (range: 0.1-48.2), and serum albumin was 3.2 mg/dl (range: 1.5-4.9). Of the 101 events, 79 (78.2%) had elevated serum creatinine levels above the normal range.

**Table 2:** Laboratory data when patients were at the consultation

		Total	No septic shock	Septic shock	*P *value
Events		101	80	21	

White blood cell count	(1,000/μL)	12.9 (0.7-38.3)	11.9 (4.2-37.7)	21.3 (0.7-38.3)	0.009

Platelet count	(10,000/μL)	14.6 (0.3-77.0)	17.0 (0.3-77.0)	8.6 (0.3-28.2)	< 0.001

Serum creatinine value	(mg/dl)	1.6 (0.5-11.6)	1.4 (0.5-11.6)	2.1 (0.5-5.1)	0.102

CRP	(mg/dl)	16.1 (0.1-48.2)	14.7 (0.1-40.2)	23.2 (2.8-48.2)	0.005

Serum albumin	(mg/dl)	3.2 (1.5-4.9)	3.2 (1.5-4.9)	2.7 (1.5-3.8)	0.017

Table 3 displays the complications and past history of the patients. Of the 101 events, 25 (24.8%) were associated with diabetes mellitus, 41 (40.6%) with hypertension, 20 (19.8%) with psychosis, and 11 (10.9%) with paralysis. Forty-two (41.6%) events were associated with a history of urolithiasis, 18 (17.8%) with cerebral infarction (or hemorrhage or aneurysm), and 12 (11.9%) with cardiovascular disease. The daily median estimated glomerular filtration rate (eGFR) was 63.0 ml/min (range: 6.7-178.0). The performance status was ≥ 2 in 27 (26.7%) events. In 64 (63.4%) of the 101 events, the criteria for sepsis were fulfilled and 21 (20.8%) met the criteria for septic shock.

**Table 3:** The characteristics of complications, past history of disease and others

	Total	No septic shock	Septic shock	*P *value
Events	101	80	21	

The complication				

Diabetes mellitus	25 (24.8%)	21 (26.3%)	4 (19.0%)	0.582

Hypertention	41 (40.6%)	34 (42.5%)	7 (33.3%)	0.618

Psychosis	20 (19.8%)	15 (18.8%)	5 (23.8%)	0.759

The presence of paralysis	11 (10.9%)	4 (5.0%)	7 (33.3%)	0.001

The past history of disease				

Urolithiasis	42 (41.6%)	36 (45.0%)	6 (28.6%)	0.215

Cerebral infarction or hemorrhage or aneurysm	18 (17.8%)	12 (15.0%)	6 (28.6%)	0.198

Cardiovascular disease	12 (11.9%)	6 (7.5%)	6 (28.6%)	0.016

Other characteristics				

Dayly renal function, median eGFR (range)	63.0 (6.7-178.0)	62.5 (6.7-131.0)	69.9 (30.0-178.0)	0.302

Performance status (The ECOG score ≧ 2)	27 (26.7%)	16 (20.0%)	11 (52.4%)	0.005

Comparison between the patients with and without septic shock showed that those with septic shock were significantly older (median: 74 years; range: 34-90 years) than those without septic shock (median: 64 years; range: 29-78 years) (*p *< 0.001) (Table 1). Patients with septic shock were significantly more likely to have bacteremia than those without septic shock (71.0% vs. 26.0%) (*p *< 0.001). In patients with septic shock, there was a significantly shorter period from the onset of symptoms to drainage (median: 2 days; range: 0-16 days) than in those without septic shock (median: 3 days; range: 0-38 days) (*p *< 0.001). Furthermore, patients with septic shock had a significantly longer hospital stay (median: 14 days; range: 1-95 days) than those without septic shock (median: 10 days; range: 1-171 days) (*p *= 0.008). Moreover, the median white blood cell count and CRP level of patients with septic shock were significantly higher than those of patients without septic shock (21.3 × 1,000/μL and 23.2 mg/dl with septic shock vs. 11.9 × 1,000/μL and 14.7 mg/dl without septic shock) (*p *= 0.009 and 0.005, respectively), while the median platelet count and serum albumin level of patients with septic shock were significantly lower (8.6 × 10,000/μL and 2.7 mg/dl with septic shock vs. 17.0 × 10,000/μL and 3.2 mg/dl without septic shock) (*p *< 0.001 and *p *= 0.017, respectively) (Table 2). Significantly more patients with septic shock had paralysis than among those without septic shock (33.3% vs. 5.0%, *p *= 0.001). Significantly more patients with septic shock also had a history of cardiovascular disease (28.6% vs. 7.5%, *p *= 0.016) and an ECOG performance status 2 (52.4% vs. 20.0%, *p *= 0.005) (Table 3). However, diabetes mellitus was not a risk factor. Multivariate analysis revealed that old age (OR: 1.07, *p *= 0.007) and the presence of paralysis (OR: 10.78, *p *= 0.004) were independent risk factors for septic shock.

## Discussion

An obstructed and infected kidney and APN are urological emergencies that sometimes progress to sepsis and/or septic shock. Despite intensive management and emergency drainage, a mortality rate of around 2% may still be expected [2]. In our series, 2 patients died of septic shock after drainage. Since acute obstructive uropathy due to upper urinary tract calculi raises the renal pelvic pressure and theoretically decreases the uptake of drugs by the kidney, sepsis due to upper urinary tract infection associated with upper tract calculi frequently requires drainage.

It has been reported that patients who are likely to develop sepsis include the elderly, as well as those with diabetes or immunosuppression (transplant recipients, patients receiving chemotherapy or corticosteroids, and patients with acquired immunodeficiency syndrome) [4]. Our analysis revealed that older age and the presence of paralysis were independent risk factors for septic shock, while a history of urolithiasis and the presence of diabetes mellitus were not associated with septic shock in our cohort. A poor performance status, as occurs in patients with spinal cord injury, is a well-known risk factor for renal calculi [5,6]. Performance status, age, and sex were reported to be independent risk factors for emergency drainage to treat APN with upper urinary tract calculi [2]. In another study, a high CRP level and old age were found to be independent predictors of the need for urinary diversion, and CRP was shown to be an objective and useful parameter for the selection of stenting in patients with renal colic [6]. Our study revealed that poor performance status was a significant risk factor for septic shock associated with upper urinary tract calculi. Elderly patients and those with paralysis often have a poor performance status. In such patients, diagnosis of APN associated with upper urinary tract calculi might be delayed because the initial symptoms are obscure or because early medical attention is not sought.

## Conclusions

APN due to ureteral obstruction by upper urinary tract calculi can be lethal despite drainage if it progresses to septic shock. Our analysis revealed that older age and the presence of paralysis were independent risk factors for septic shock in patients who required emergency drainage. Prevention of urolithiasis and urinary tract infection in elderly individuals and patients with paralysis could be of considerable importance. Moreover, careful treatment is required for patients with APN and upper urinary tract calculi, particularly those with risk factors for septic shock.

## Competing interests

The authors declare that they have no competing interests.

## Authors' contributions

YY, KF, SN, TH, GT, RI, MH, DW, SF and SY identified patient cases and assisted in the acquisition of data. YY, KF assisted in the analysis and interpretation of data, and prepared the initial manuscript. All authors revised the manuscript and all have read and approve of the final manuscript.

## Pre-publication history

The pre-publication history for this paper can be accessed here:

http://www.biomedcentral.com/1471-2490/12/4/prepub
